# Prevalence of Insulin Resistance in Type 1 Diabetes Mellitus and Its Correlation with Metabolic Parameters: The Double Trouble

**DOI:** 10.5152/eurasianjmed.2022.21039

**Published:** 2022-06-01

**Authors:** Vaibhav Pathak, Ipsita Mishra, Anoj Kumar Baliarsinha, Arun Kumar Choudhury

**Affiliations:** 1 Department of Endocrinology, SCB Medical College and Hospital, Cuttack, Odisha, India

**Keywords:** Double diabetes, estimated glucose disposal rate, insulin resistance, type 1 diabetes mellitus, waist circumference

## Abstract

**Objective:** The phenotype of type 1 diabetes mellitus has changed over the last few decades. Little attention has been paid to the presence of insulin resistance in individuals with type 1 diabetes mellitus. The appearance of insulin resistance in type 1 diabetes mellitus patients has been labeled as “double diabetes.” This phenotype of double diabetes has been seen to have higher rate of microvascular as well as macrovascular complications. The aim of the current study was to estimate the burden of insulin resistance in patients with type 1 diabetes mellitus and its correlation with various metabolic parameters and microvascular complications.

**Materials and Methods:** It was a cross-sectional study in which a total of 95 type 1 diabetes mellitus patients (children/adolescents (<18 years) and adults ≥18 years) presenting to Endocrinology OPD were screened for the presence of insulin resistance using estimated glucose disposal rate. Estimated glucose disposal rate (mg/kg/min) was calculated as = 21.16− (0.09 ×WC) − (3.407×HTN) − (0.551×HbA1c [%]) where, WC is waist circumference (cm) and HTN is hypertension (1= yes, 0 = no). Based on previous studies, an estimated glucose disposal rate <8 was considered to have the presence of insulin resistance and double diabetes.

**Results:** Using an estimated glucose disposal rate <8 as the cut-off for the presence of insulin resistance, the overall prevalence was 16.8%. Prevalence was high in adults 12 (29.3%) compared to children/adolescents 4 (7.4%) which was statistically significant [χ^2^ = 7.95; *P* = .004]. In comparison of the anthropometric and metabolic parameters in those with an estimated glucose disposal rate <8 versus ≥8, there was a significant statistical difference. Those having an estimated glucose disposal rate <8 had higher age, longer duration of diabetes, and body mass index [*P* ≤ .05]. Also, they had poor glycemic control, higher blood pressure, triglycerides, low-density lipoproteins levels. Using Spearman correlation coefficient there was a statistically significant (*P* < .05) negative correlation between the estimated glucose disposal rate and various anthropometric as well as metabolic parameters.

**Conclusion:** This study shows that with increasing duration of disease, insulin resistance (low estimated glucose disposal rate) could be a serious problem in type 1 diabetes mellitus patients, especially in those who are metabolically unhealthy. As insulin resistance could be a major contributing factor in the onset and progression of various vascular complications, evaluation of the presence of insulin resistance using estimated glucose disposal rate could be useful in recognizing individuals who would benefit the most from preventative strategies.

**Main Points**

- With the increasing prevalence of obesity across the world insulin resistance in type 1 diabetes mellitus (T1D) subjects is of concern.
- Insulin resistance in T1D subjects could be the main driver of the rapid progression of vascular complications.
- Estimated glucose disposal rate is a simple tool that can be easily used to identify those at risk.
- Aggressive lifestyle and behavior modifications as well as treatment of modifiable risk factors should be considered at the earliest possible time.

## Introduction

Type 1 diabetes mellitus (T1D) has been generally characterized as a condition with an insulin-deficient phenotype. The presence of insulin resistance (IR) in patients with T1D, however, has led to the appearance of a different phenotype of mixed T1D and type 2 diabetes mellitus (T2D), or “double diabetes” (DD).^1^ This phenotype of DD has been associated with an increased rate of microvascular complications as well as cardiovascular diseases in individuals with T1D.^2,3^ Despite the phenotype of DD known for decades, there is still no standard criteria to define this set of individuals with T1D.^4^ Focus while defining DD has been mainly on the presence of obesity, hypertension (HTN), dyslipidemia, and family history of T2D.^5,6^

The gold standard to measure IR is the euglycaemic-hyperinsulinemic clamp, but, being invasive and time consuming, it is not feasible for routine use.^7^ Estimated glucose disposal rate (eGDR) has been suggested as a surrogate method for measurement of IR that is easy to use. The eGDR equation was devised and validated using clamp studies in a subset of T1D patients in which lower values suggested higher IR.^8^ They proposed that eGDR can be calculated using clinical parameters including HTN, glycated hemoglobin (HbA1c) and waist–hip ratio or body mass index (BMI), or waist circumference (WC).^8,9^

Although eGDR is similar to metabolic syndrome (Met S) in incorporating WC and blood pressure in score, yet, it is a continuous variable, making it alluring for routine use. This is of significance because a decline in eGDR with time has been associated with a higher risk of atherosclerotic cardiovascular diseases and death.^10-13^ Other studies not only provide support for the association between low eGDR and high risk of vascular complications but also showed the upper hand of eGDR in predicting complications compared with the use of Met S to define DD.^14^

Estimated glucose disposal rate has shown to be a useful parameter to pick out DD, but the cut-off value needs cautious consideration. Nyström et al^15^ in a study from Sweden showed an eGDR <8 mg/kg/min was associated with high cardiovascular risk compared to those with eGDR ≥8 mg/kg/min and the risk was markedly high among those with the lowest eGDR values. Hermosillo et al^1^ showed that an eGDR value <7.32 mg/kg/min had the highest sensitivity and specificity to detect Met S in patients with T1D. Hence, the eGDR value of <8 seems to be convincingly useful to identify patients with DD among T1D, with a higher risk in those with the lowest eGDR.

To the best of our knowledge, there is no study from India, which has tried to look into the prevalence of IR in T1D subjects using eGDR. Thus, the aim of the present study was to assess IR in subjects with T1D by eGDR estimation and its correlation between clinical and biochemical parameters.

## Materials and Methods

It was a cross-sectional study in which total 95 T1D patients (children/adolescents and adults) attending the outpatient Endocrinology clinic were recruited. Informed consent was obtained from patients (who were more than 18 years) or their guardians (those who were less than 18 years old) involved in the study. History and physical examination of all patients were done using the case proforma. Patients needed to have at least a minimum of 5 years duration of diabetes before being recruited for the study. A single investigator registered the weight (kilograms), height (centimeters), WC (centimeters), blood pressure, and calculated BMI (kg/m^2^). A direct interview was done for family history of T2D, cardiovascular risk factors, and the presence of diabetes complications. Microvascular complication screening was performed by a single investigator in which diabetic retinopathy was defined as fundoscopic changes suggestive of mild non-proliferative change or more. The presence of neuropathy was defined as impaired 10 g monofilament testing. Urine collection to assess the albumin to creatinine ratio (mg/g) was done and a previous report was looked at in those with hyperglycemia. The HbA1c was measured by high-performance liquid chromatography, with a Bio-Rad D10 analyzer (Bio-Rad Laboratories, CA, USA) and the coefficient of variation was 1.8%. HbA1c measurement was standardized, conformed to National Glycosylated Hemoglobin Standardization Program, and aligned to Diabetes Control and Complications Trial assay. eGDR (mg/kg/min) was calculated as eGDR = 21.16− (0.09 ×WC) − (3:407×HTN) − (0.551×HbA1c [%]). Based on previous studies an eGDR<8 was considered to be suggestive of the presence of IR.^1,15^

### Ethical Clearance

The ethical clearance for the study was obtained from SCB Medical College Utkal University (884/14-10-2019).

### Statistical Analysis

Descriptive statistical methods such as mean and standard deviation will be applied to summarize continuous variables. Categorical data will be summarized as percentages and proportions. An unpaired *t*-test was used for comparison of the mean of 2 groups. The normality distribution of all parameters was checked using Shapiro–Wilk test. Non-parametric tests (Mann–Whitney *U* test) and parametric tests (independent *t*-tests) were performed to compare between parameters as required. For correlation between parameters Spearman (non-parametric) and Pearson (parametric) correlation coefficients were. For associations between eGDR and microvascular complications, odds ratio (OR) was calculated. All statistical analysis was done using SPSS21.

## Results

**Figure 1.**
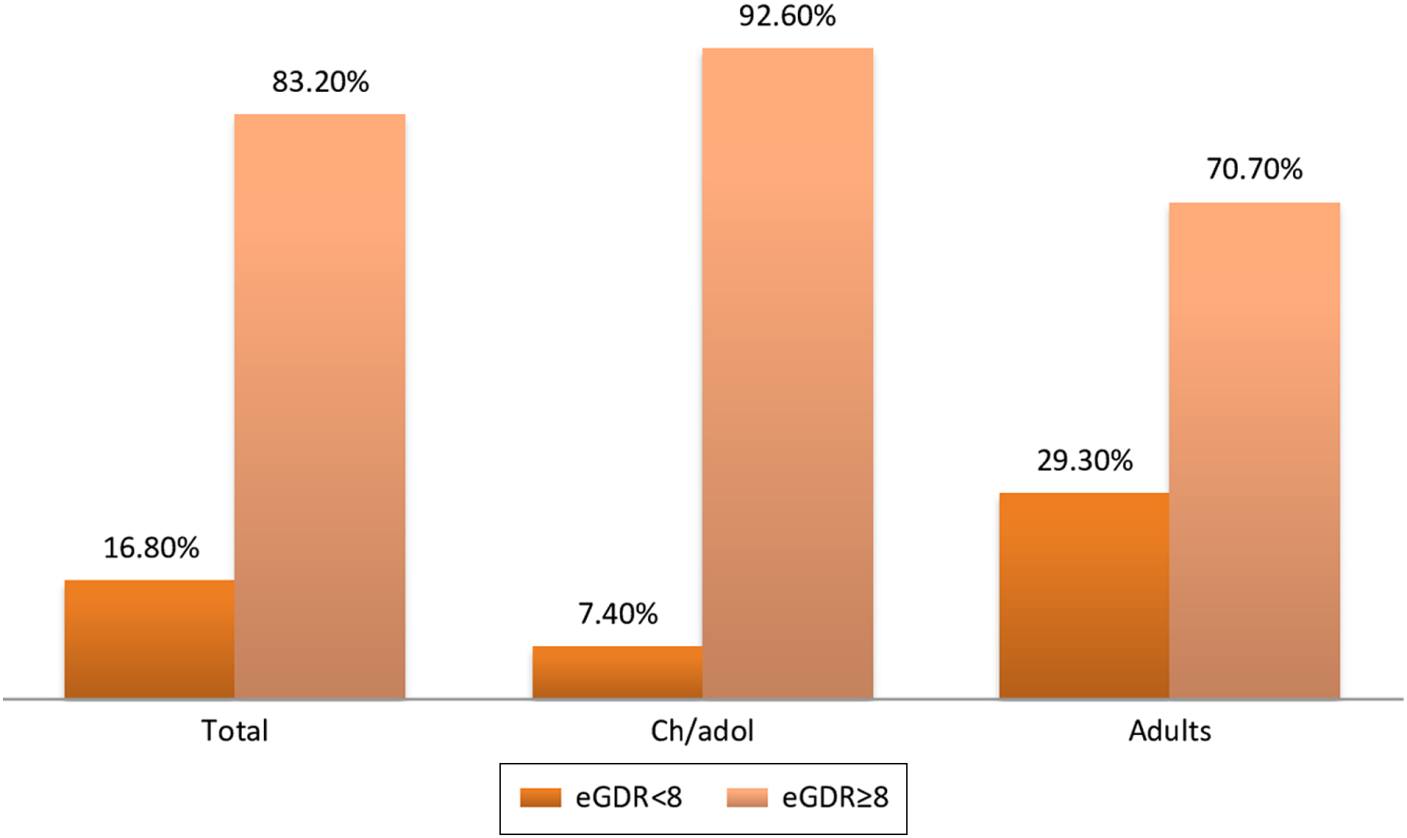
Prevalence of IR in children/adolescent and adults. IR, insulin resistance.

There were a total of 95 patients out of which 54 were children/adolescents and 41 were adults. The mean age in children/adolescents and adults was 13.31 ± 2.718 and 24.39 ± 6.64, respectively. Among children/adolescents, 24 were males and 30 were females while in adults, 28 were males and 13 were females. Mean BMI and WC in children/adolescents were (18.62 ± 2.20; 66.07 ± 7.402) while in adults they were (21.66 ± 2.32; 75.51 ± 7.96) (Table 1). The mean duration of diabetes was 5.98 ± 1.25 years in children/adolescents while it was 8.71 ± 5.40 years in adults. Mean eGDR was (10.02 ± 1.39) in children/adolescents while it was (9.00 ± 1.99) in adults (Table 1). Total 16 (16.8%) patients had eGDR <8. Estimated glucose disposal rate <8 was present in 4 (7.4%) of children/adolescents and in 12 (29.3) of adults which were statistically significant [χ^2^ = 7.95; *P* = .004] (Figure 1). In comparison of the anthropometric and metabolic parameters in those with Egdr <8 versus ≥8, there was A significant statistical difference between the 2. Those having eGDR <8 had higher age, longer duration of diabetes, high WC, insulin dose, and BMI. Also, they had poor glycemic control, higher systolic blood pressure, diastolic blood pressure, triglyceride (TG), and low-density lipoprotein (LDL) levels (Table 2). Various parameters in those with eGDR <8 versus ≥8 were compared in children/adolescents and adults individually and it showed statistical significance more in adults (Tables 3 and 4). Using Spearman correlation there was a significant negative correlation between eGDR values and various anthropometric as well as metabolic parameters (Table 5). Total 25 patients (26.3%) had microvascular complications of which albuminuria was most common (n = 24; 25.3%) followed by neuropathy (n = 17; 17.9%) and retinopathy (n = 11; 11.6%). None of the subjects had a history of any form of macrovascular event. We also estimated the association of microvascular complications with eGDR. Odds ratio for low eGDR association with overall microvascular complication was 5.063 [95% CI (1.63-15.67)], albuminuria was 3.93 [95% CI (1.28-12.10)], neuropathy was 7.77[95% CI (2.34-25.84)] and retinopathy was 14.58 [95% CI (3.56-59.71)] (Table 6).

## Discussion

The phenotype of T1D has changed during the last few decades and increasingly Met S and its components are being reported in individuals with T1D [16]. Little clinical attention has been paid to IR as a common feature of T1D patients. The growing obesity prevalence in the T1D population will further lead to an increase in IR, an important clinical factor associated with vascular complications. Lack of physical activity coupled with nutrient-poor high caloric food is leading to an increasing trend of obese subjects with T1D.^16^ The challenge for clinicians is to be vigilant about the subset of patients who are at increased risk of slipping into the DD category. Moreover, individuals with T1D itself usually have a lifetime exposure to diabetes mellitus which itself is an independent cardiovascular risk factor, and further development of IR could act as fuel to fire leading to devastating consequences.

Using eGDR <8, the prevalence of DD in T1D subjects was 16.8% in our study. However, it was much higher in adults (29.3%) compared to children/adolescents (7.4%) which was significant statistically (χ^2^ = 7.95; *P* = .004). Nyström et al^15^ using eGDR <8 reported the prevalence of DD in T1D to be 51%, which was higher as compared to our results. This difference could probably be because of the large sample, the higher mean age of the study population, and the longer duration of diabetes in their study. However, in a study by Merger et al^17^ from Germany, the prevalence of DD was 25.5% in adults which was similar to our results in adults. Chillarón et al^18^ in a study from Spain reported a prevalence of 31.9% which is also similar to our results. These results and as well as findings from this study suggest that IR could be common in T1D subjects. In contrast to knowledge about IR in adults, there have been very few studies about the use of eGDR in children. In a study by Palomo et al^9^ in children, the mean value of eGDR was significantly lower in obese subjects and they suggested that eGDR could be a useful marker of IR in children as well. Mishra et al^20^ reported a 7% prevalence of DD in youth which is similar to 7.4% in this study.

Studies have used obesity or Met S to define DD prevalence but a caveat when using them as sole criteria for DD prevalence estimation may be that although being easy to use but it is likely to miss a number of patients with DD, and therefore more explicit measures are needed for which eGDR fits the bill.^1,16^

Type 1 diabetes mellitus patients who are unhealthy metabolically are the ones to form the core group with DD as was shown in this study. Compared to T1D subjects with eGDR≥8, those with eGDR <8 had higher BMI, age, WC, poor glycemic control, TG, and LDL. The pathogenic ingredients for the development of IR are hereditary predisposition and environmental risk factors. These can combine with T1D duration, making the development of IR a time-dependent condition, as shown in this study that those with DD phenotype had a longer duration of diabetes. While the hereditary component is non-modifiable, environmental risk factors can be mitigated, thus reducing the development of DD. The obesogenic environment does contribute to the development of overweight and obesity in T1D but cannot be held solely responsible. Because more individuals with T1D are overweight and obese than those without diabetes, indicating the presence of additional contributing mechanisms.^21,22^ Delivery of subcutaneous insulin in a non-physiological manner along with abnormal eating behavior in fear of hypoglycemia could be additional factors in the development of DD.^23,24^ Therefore, the development of IR in T1D subjects is secondary to a combination of unhealthy lifestyle choices as well as added diabetes-specific mechanisms.

Studies have shown that with a reduction in HbA1c, there is a decrease in microvascular complications and long-term macrovascular disease.^25^ However, there is great heterogeneity in the rate of occurrence of complications, indicating that factors other than HbA1c do have a role. Merger et al^17^ in their study found that subjects with DD had a markedly higher rate of micro- and macrovascular complications, which persisted even after adjustments for various factors. An alarming finding was that the rate of complications was higher in the DD subgroup regardless of glycemic control suggesting that poor glycemic control is not the sole factor responsible for poor outcomes in T1D patients. In this study subjects who had eGDR <8, had higher odds of developing microvascular complications compared to those with eGDR ≥8. Although we did not evaluate for macrovascular complications, negative correlation of anthropometric and metabolic parameters with eGDR showed a trend toward adverse metabolic profile in those with low eGDR. Epstein et al^9^ showed similar results that patients with the eGDR values in the lowest quartile compared with the highest had a significantly higher risk of any diabetes complication (OR 3.1 [95% CI (1.2–8.1)]). Chillarón et al^19^ in a study from Spain also showed similar findings that patients with diabetes-related complications had low eGDR values.

Although eGDR is easy to use for risk stratification of T1D patients, it should be used with caution. Different studies have proposed different thresholds for risk stratification (range being 6-8).^1,15,18^ eGDR values may differ by ethnicity as well.^9^ There is no denying that a population-specific cut-off is needed and would be ideal to use. Nonetheless, clinicians can use eGDR in routine clinical practice to be cautious about future metabolic risks. A simple strategy could be to use eGDR as a routine clinical tool for screening T1D patients, and those with low eGDR or progressively decreasing eGDR during follow-up, should be aggressively managed for metabolic parameters. This could help in reducing the morbidity and mortality in T1D patients.

A few strengths of our study are to the best of our knowledge this is the first study from India to use eGDR for estimating the prevalence of DD in the T1D population. The limitations are the cross-sectional nature of the current study. So, no conclusions regarding causality can be drawn. Also, eligibility for inclusion was based on a clinical diagnosis of T1D, and confirmatory laboratory indicators (e.g., anti-GAD antibody) were available for only a subset of patients. As the mean duration of diabetes was less in our study compared to other studies we could not comment on macrovascular events.

Future longitudinal studies are needed to delineate the relationship between the progressive decline in eGDR and the development of vascular complications in subjects with T1D. Studies are also needed to determine whether therapies to reduce IR can avoid vascular damage in these patients.

In conclusion, this study corroborates with previous studies that with increasing duration of disease, IR (low eGDR) could be a serious problem in T1D patients, especially in those who are metabolically unhealthy. Estimation of eGDR could be useful in identifying subjects who might benefit the most from early preventative strategy.

## Figures and Tables

**Table 1.** Various Characteristics of Children/Adolescents and Adults

	Children/Adolescents, N = 54 (Mean ± SD)	Adults, N = 41 (Mean ± SD)
Age (years)	13.31 ± 2.718(Min-6) (Max-17)	24.39 ± 6.64(Min-18) (Max-50)
Gender	Male-24 Female-30	Male-28 Female-13
BMI (kg/m^2^)	18.62 ± 2.20	21.66 ± 2.32
Duration of DM (years)	5.98 ± 1.25	8.71 ± 5.40
Waist circumference (cm)	66.07 ± 7.402	75.51 ± 7.96
Insulin dose (IU/kg/day)	0.94 ± 0.14	1.03 ± 0.20
SBP (mmHg)	100.22 ± 8.83	118.92 ± 8.90
DBP (mmHg)	63.22 ± 6.01	72.43 ± 8.40
HbA1c (%)	9.40 ± 2.18	9.27 ± 2.21
TG (mg/dL)	132.00 ± 40.08	150.10 ± 33.72
CH (mg/dL)	154.83 ± 25.06	181.95 ± 26.96
LDL (mg/dL)	90.41 ± 22.67	113.25 ± 22.95
HDL (mg/dL)	38.02 ± 6.60	38.68 ± 7.64
VLDL (mg/dL)	26.40 ± 8.01	30.02 ± 6.74
eGDR (mg/kg/min)	10.02 ± 1.39	9.00 ± 1.99

SBP**,** systolic blood pressure; DBP, diastolic blood pressure; HbA1c, glycated hemoglobin; TG, triglyceride; CH, Total Cholesterol; LDL, low-density lipoproteins; HDL, high-density lipoproteins; VLDL, very low-density lipoproteins; Egdr, estimated glucose disposal rate; DM, diabetes mellitus; BMI, body mass index.

**Table 2.** Comparison of Various Characteristics of Patients with eGDR <8 and eGDR ≥8

	eGDR<8 [N = 16] (Mean ± SD)	eGDR ≥8 [N = 79] (Mean ± SD)	*P*
Age (years)	24.00 ± 10.60	16.90 ± 5.83	<.01
BMI (kg/m^2^)	21.43 ± 3.00	19.63 ± 2.55	<.01
Duration of DM (years)	10.63 ± 7.77	6.46 ± 1.90	.05
Insulin dose (U/kg/day)	1.32 ± 0.10	0.91 ± 0.09	<.01
WC (cm)	78.44 ± 9.12	68.47 ± 7.96	<.01
HbA1c (%)	12.69 ± 2.34	8.67 ± 1.38	<.01
TG (mg/dL)	152.63 ± 34.87	137.22 ± 38.71	.144
CH (mg/dL)	189.81 ± 32.42	161.82 ± 26.14	<.01
LDL (mg/dL)	120.54 ± 30.87	96.16 ± 22.15	<.01
HDL (mg/dL)	38.75 ± 7.12	38.22 ± 7.06	.783
VLDL (mg/dL)	30.52 ± 6.97	27.44 ± 7.74	.144
SBP (mmHg)	120.37 ± 13.64	105.84 ± 11.23	<.01
DBP (mmHg)	77.00 ± 9.54	65.21 ± 6.70	<.01

SBP**,** systolic blood pressure; DBP, diastolic blood pressure; HbA1c, glycated hemoglobin; TG, triglyceride; CH, Total Cholesterol; LDL, low-density lipoproteins; HDL, high-density lipoproteins; VLDL, very low-density lipoproteins; WC, waist circumference; DM, diabetes mellitus; BMI, body mass index.

**Table 3.** Comparison of Various Characteristics of Patients with eGDR <8 and eGDR ≥8 in Children and Adolescents

	eGDR <8 [N = 4] (Mean ± SD)	eGDR≥8 [N = 50] (Mean ± SD)	*P*
Age (years)	14.00 ± 1.82	13.26 ± 2.78	.605
BMI (kg/m^2^)	18.05 ± 1.81	18.67 ± 2.23	.590
Duration of DM (years)	6.00 ± 0.816	5.98 ± 1.28	.976
Insulin dose (U/kg/day)	1.33 ± 0.10	0.91 ± 0.09	<.01
WC (cm)	71.00 ± 4.83	65.68 ± 7.46	.110
HbA1c (%)	15.08 ± 1.74	8.95 ± 1.45	<.01
TG (mg/dL)	142.50 ± 52.43	131.16 ± 39.49	.699
CH (mg/dL)	153.25 ± 37.87	154.96 ± 24.32	.934
LDL (mg/dL)	85.50 ± 37.80	90.81 ± 21.60	.799
HDL (mg/dL)	39.25 ± 6.75	37.92 ± 6.65	.702
VLDL (mg/dL)	28.50 ± 10.48	26.23 ± 7.90	.699
SBP (mmHg)	104.50 ± 5.74	99.88 ± 8.98	.319
DBP (mmHg)	69.50 ± 3.41	62.72 ± 5.90	.029

SBP**,** systolic blood pressure; DBP, diastolic blood pressure; HbA1c, glycated hemoglobin; TG, triglyceride; CH, Total Cholesterol; LDL, low-density lipoproteins; HDL, high-density lipoproteins; VLDL, very low-density lipoproteins; WC, waist circumference; DM, diabetes mellitus; BMI, body mass index.

**Table 4.** Comparison of Various Characteristics of Patients with eGDR <8 and eGDR ≥8 in Adults

	eGDR <8 [N = 12] (Mean ± SD)	eGDR ≥8 [N = 29] (Mean ± SD)	*P*
Age (years)	27.33 ± 10.19	23.17 ± 4.12	.067
BMI (kg/m^2^)	22.56 ± 2.41	21.28 ± 2.22	.11
Duration of DM (years)	12.17 ± 8.48	7.28 ± 2.47	<.01
Insulin dose (U/kg/day)	1.32 ± 0.10	0.91 ± 0.08	<.01
WC (cm)	80.92 ± 8.95	73.28 ± 6.43	<.01
HbA1c (%)	11.90 ± 1.97	8.18 ± 1.12	<.01
TG (mg/dL)	156.00 ± 29.30	147.66 ± 35.58	.478
CH (mg/dL)	202.00 ± 19.48	173.66 ± 25.30	<.01
LDL (mg/dL)	132.22 ± 17.73	105.40 ± 20.27	<.01
HDL (mg/dL)	38.50 ± 7.52	38.72 ± 7.81	.958
VLDL (mg/dL)	31.20 ± 5.86	29.53 ± 7.11	.478
SBP (mmHg)	125.66 ± 11.08	116.13 ± 6.11	<.01
DBP (mmHg)	79.50 ± 9.69	69.51 ± 5.82	<.01

SBP, systolic blood pressure; DBP, diastolic blood pressure; HbA1c, glycated hemoglobin; TG, triglyceride; CH, Total Cholesterol; LDL, low-density lipoproteins; HDL, high-density lipoproteins; VLDL, very low-density lipoproteins; WC, waist circumference; DM, diabetes mellitus; BMI, body mass index.

**Table 5.** Correlation of eGDR with Anthropometric and Metabolic Parameters

**Parameters**	**Correlation Coefficient**	*P*
Age (years)	−0.344	.001
Duration of DM (years)	−0.267	.009
BMI (kg/m^2^)	−0.324	.001
Insulin Dose (U/kg/day)	−0.588	.001
Weight (kg)	−0.331	.001
Family history of T2DM	−0.563	.001
TG (mg/dL)	−0.293	.004
Total cholesterol (mg/dL)	−0.376	.001
HDL (mg/dL)	−0.096	.353
LDL (mg/dL)	−0.325	.001
VLDL (mg/dL)	−0.293	.004

TG, triglyceride; LDL, low-density lipoproteins; HDL, high-density lipoproteins; VLDL, very low-density lipoproteins; DM, diabetes mellitus; BMI, body mass index.

**Table 6.** Association of low eGDR with Various Microvascular Complications

**eGDR** (≤8 low eGDR)	**OR**	**95% [CI]**
Microvascular complications present	5.063	[1.63-15.67]
Albuminuria	3.93	[1.28-12.10]
Retinopathy	14.58	[3.56-59.71]
Neuropathy	7.77	[2.34-25.84]

OR, odds ratio.
